# Association between dietary antioxidant levels and diabetes: a cross-sectional study

**DOI:** 10.3389/fnut.2024.1478815

**Published:** 2024-10-23

**Authors:** Lei Zhou, Xiaoyu Xu, Yize Li, Shuo Zhang, Hong Xie

**Affiliations:** School of Public Health, Bengbu Medical University, Bengbu, China

**Keywords:** diabetes mellitus, composite dietary antioxidant index, dietary antioxidant, oxidative stress, NHANES

## Abstract

**Background:**

The onset and progression of diabetes mellitus (DM) is strongly linked to oxidative stress. Previous studies have highlighted the protective effects of individual dietary antioxidants against diabetes. However, the relationship between a comprehensive combination of dietary antioxidants and diabetes has rarely been examined. Therefore, this study assessed the association between various dietary antioxidant intake levels and diabetes among US adults and further investigated potential associations using the Composite Dietary Antioxidant Index (CDAI).

**Methods:**

The study employed data from the National Health and Nutrition Examination Survey (NHANES) conducted between 2011 and 2018 for cross-sectional analysis. Dietary information was obtained from two 24-h dietary recall interviews. The CDAI was calculated using intakes of six dietary antioxidants from the dietary information. Multifactorial logistic regression models were employed to investigate the association of different dietary antioxidants and CDAI with DM. The relationship between CDAI and DM was further explored using subgroup analyses and restricted cubic spline curves.

**Results:**

A total of 7,982 subjects (mean age 47.32 ± 16.77 years; 48.50% male and 51.50% female) were included in this study. In the multivariate-adjusted single antioxidant model, vitamin C intake was significantly and negatively associated with diabetes prevalence (P for trend = 0.047), while zinc intake demonstrated a potential trend toward reduced diabetes risk (P for trend = 0.088). This association was similarly observed in the multivariate-adjusted model for the Composite Dietary Antioxidant Index (CDAI) in the female population (*p* = 0.046).

**Conclusion:**

Intake of vitamin C was negatively associated with DM prevalence. Additionally, CDAI was found to reduce the risk of DM in the female population.

## 1 Introduction

The high prevalence of type 2 diabetes has emerged as a critical public health issue worldwide (1). The global prevalence of type 2 diabetes mellitus (T2DM) in adults has surged from approximately 150 million in 2000 to 450 million in 2019, and projections suggest it will rise to around 700 million by 2045 (2). In 2017, the total economic burden of diagnosed diabetes in the United States was $327 billion, with patient care accounting for 24% of all healthcare costs (3). This underscores the urgency of diabetes prevention and treatment.

Oxidative stress is intricately involved in the onset and progression of type 2 diabetes, significantly contributing to this process. Insulin resistance and impaired beta-cell function are often present in the prodromal stages of diabetes. Elevated oxidative stress can exacerbate insulin resistance and impair insulin secretion. Furthermore, oxidative stress increases the incidence of diabetic complications. Elevated levels of reactive oxygen species (ROS) and reactive nitrogen species (RNS) are associated with lipid peroxidation, non-enzymatic glycation of proteins, and glucose oxidation, all of which promote diabetes and its complications (4, 5).

Evidence is mounting that certain dietary antioxidants, such as vitamins C and E, and carotenoids, may reduce the risk of developing type 2 diabetes (6–8). However, the relationship between the intake of vitamin A, zinc, and diabetes remains contentious (9, 10). One study reported no significant correlation between total or supplemental zinc intake and type 2 diabetes mellitus (T2DM) (11). Conversely, another study indicated that sufficient vitamin A intake might help prevent diabetes, particularly in men (12). The association between selenium levels and diabetes risk is also inconclusive (13).

Diets typically contain various antioxidants that may have synergistic or additive effects (14). The Composite Dietary Antioxidant Index (CDAI) reflects an individual’s overall antioxidant capacity, with higher scores indicating greater capacity (15). Previous studies have reported that a high CDAI is associated with a reduced prevalence of chronic diseases like chronic kidney disease (CKD) and chronic obstructive pulmonary disease (COPD) (16, 17). However, epidemiological evidence linking CDAI and diabetes is limited, with only one prior study examining this relationship (18).

This study aims to investigate the association between different dietary antioxidant levels and DM among adult participants in the National Health and Nutrition Examination Survey (NHANES) database. It further explores the potential association between CDAI and DM, contributing to the existing body of knowledge in this field.

## 2 Materials and methods

### 2.1 Data sources

National Health and Nutrition Examination Survey (NHANES) is a large-scale, population-based, cross-sectional survey designed to assess the health and nutritional status of adults and children in the United States. This unique survey integrates interviews with physical examinations, covering demographics, diet, blood biochemistry, and more, with new data sets released biennially (19, 20).

### 2.2 Study population

**Figure 1:**
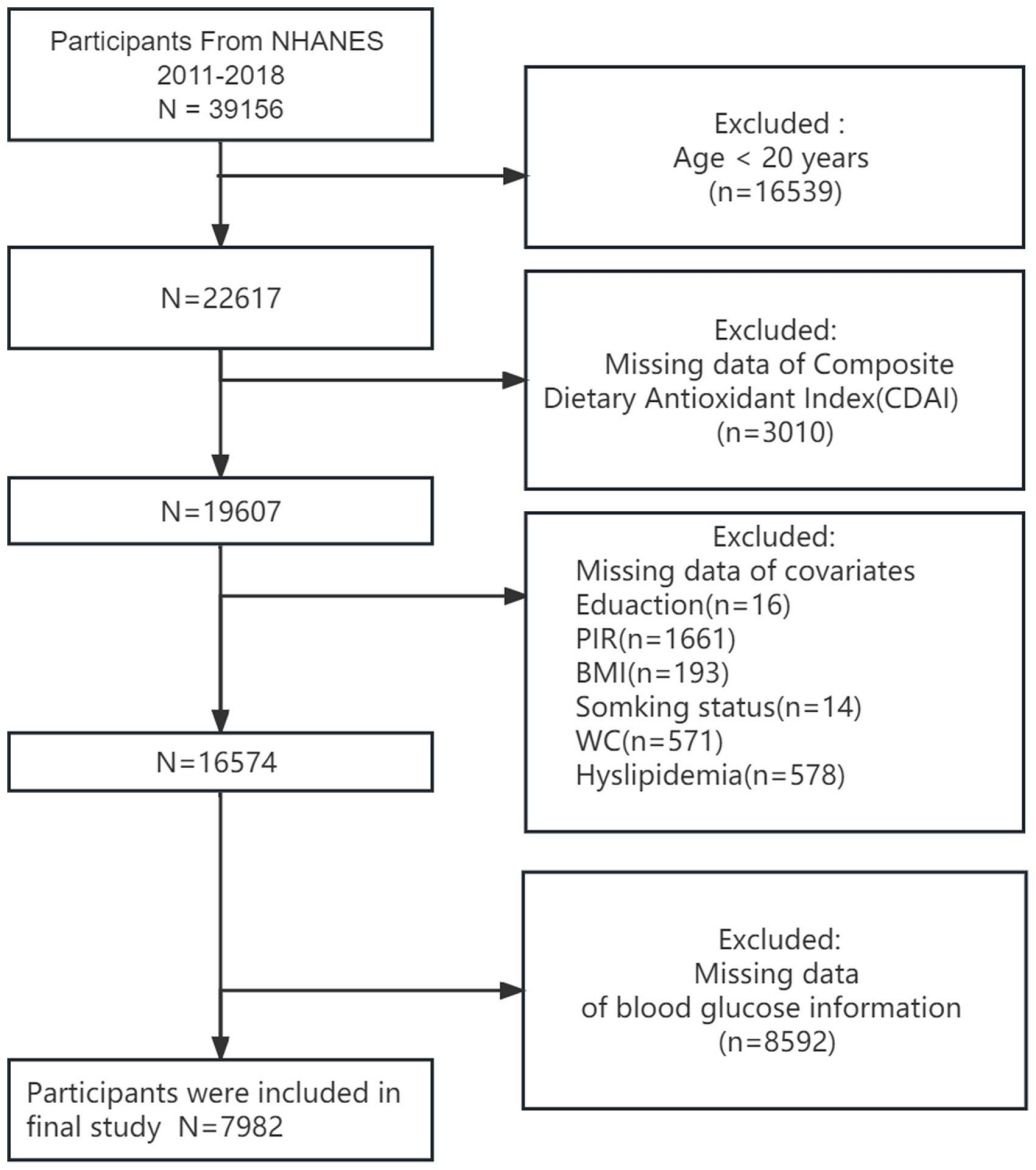
A flowchart showing the selection of study participants.

From 2011 to 2018, encompassing four NHANES cycles, 39,156 participants completed the survey. We excluded 16,539 participants under 20 years of age and 3,010 participants without dietary information. Additionally, we excluded 16 participants lacking education information, 14 without smoking status, 1,661 without a poverty index, 193 without body mass index data, 105 without waist circumference data, and 8,592 participants without blood glucose information. Consequently, a total of 7,982 participants were eligible for this study (Figure 1). These data were sourced from the official NHANES website.

### 2.3 Diet assessment

Detailed dietary intake information was obtained from NHANES participants through the dietary interview component. All NHANES participants were eligible for two 24-h dietary recall interviews. The first dietary recall interview was conducted in person at a mobile examination center (MEC), and the second interview was conducted by telephone 3 to 10 days later. The Food and Nutrient Database for Dietary Studies (FNDDS) is used to process dietary information for each cycle, including the composition and total amount of various nutrients contained in individual foods and beverages.

The Composite Dietary Antioxidant Index (CDAI) was calculated from the mean dietary intake of vitamin A, vitamin C, vitamin E, zinc, selenium, and carotenoids obtained from the two 24-h recalls. A standardization of each antioxidant (x_i_) was performed by subtracting the gender-specific mean (μ_i_) and dividing by the gender-specific standard deviation (s_i_) (21). Please refer to the following equations:


$$CDAI=\overset{6}{\underset{i=1}{\sum }}\frac{X_{i}-\mu_{i}}{s_{i}}$$

### 2.4 Ascertainment of covariates

In accordance with previous literature and theoretical considerations, we included the following variables as potential confounders in the study: age, gender, race, education level, poverty-to-income ratio (PIR), body mass index (BMI), physical activity, smoking status, daily alcohol intake, total daily energy intake, hypertension, and dyslipidemia. BMI was calculated by dividing body weight (kilograms) by the square of height (meters). Race was categorized into Non-Hispanic White, Non-Hispanic Black, Mexican American, and other groups. Educational background was classified as less than high school (<9th grade or grades 9–11, including 12th grade without a diploma), high school graduate/GED or equivalent, and more than high school (some college, associate’s degree, or college graduation or above) (16).

Activity status was categorized as high (any high-intensity exercise, fitness, or recreational activity resulting in a substantial increase in respiration or heart rate within 1 week), moderate (any moderate-intensity exercise, fitness, or recreational activity resulting in a slight increase in respiration or heart rate within 1 week), and low (not meeting the above criteria). Hypertension was defined as a mean systolic blood pressure (SBP) ≥140 mmHg and/or mean diastolic blood pressure (DBP) ≥90 mmHg, self-reported diagnosis of hypertension, or use of antihypertensive medication (22) Dyslipidemia was defined as either a serum total cholesterol level > 251 mg/dL (6.5 mmol/L) or use of lipid-lowering drugs (23). Data and measurements were obtained from NHANES.

### 2.5 Ascertainment of outcomes

Referring to the recommendations of the American Diabetes Association (ADA) (24), type 2 diabetes was defined by any of the following criteria: (1) physician-diagnosed diabetes mellitus, (2) fasting blood glucose level ≥ 126 mg/dL (7.0 mmol/L), (3) glycosylated hemoglobin HbA1c ≥ 6.5% (48 mmol/mol), or (4) use of glucose-lowering medications or insulin.

### 2.6 Statistical analysis

Continuous variables are presented as weighted means ± standard deviations, while categorical variables are shown as unweighted counts (weighted percentages). Comparisons between categorical variables were conducted using the chi-square test. Multivariate logistic regression analyses explored the association between dietary antioxidant intake (vitamins A, C, and E; carotenoids; selenium; and zinc) and CDAI with the prevalence of diabetes mellitus (DM). The analyses were adjusted as follows: Model 1 was unadjusted, Model 2 adjusted for age, sex, race, and education, and Model 3 adjusted for all potential covariates. Dietary antioxidant intake and CDAI were divided into quartiles (Q1, Q2, Q3, and Q4), and *p*-values for trends were calculated. Multivariate-adjusted restricted cubic spline (RCS) curves were used to explore the nonlinear relationship between CDAI and diabetes. Further stratified analyses examined the association between CDAI and DM by age (<60 years/≥60 years), sex (male/female), BMI (<25.0/25.0–30.0/≥30.0), PIR (<1.3/1.3–3.2/≥3.2), hypertension (yes/no), and dyslipidemia (yes/no). Data were weighted to ensure the analysis was representative of the general population. All statistical assumptions were verified at a significance level of 0.05. Analyses were conducted using R version 4.40.

## 3 Results

### 3.1 Participant characteristics

A total of 7,982 subjects (mean age 47.32 ± 16.77 years; 48.50% male, 51.50% female) were included, of whom 1,607 were diabetic and 6,375 were non-diabetic. Table 1 presents the weighted baseline characteristics of the study population. Age, race, education level, PIR, BMI, physical activity, smoking status, daily alcohol intake, total daily energy intake, hypertension, and dyslipidemia differed significantly between groups (*p* < 0.05). The non-DM group had lower age, BMI, glucose, and HbA1c levels, but higher PIR, daily alcohol consumption, total daily energy intake, and CDAI scores. A greater proportion of individuals in the DM group had hypertension (68.63%) and dyslipidemia (54.64%). Gender differences were not significant (*p* > 0.05).

**Table 1:** General characteristics of participants (*N* = 7,982) stratified by DM or non-DM in the NHANES 2011–2018 data.

Characteristic	Overall	Non-DM	DM	*P*-value
Age [mean (SD)]	47.32 ± 16.77	45.36 ± 16.50	58.57 ± 13.57	<0.001
Gender (%)				0.053
Female	4,077 (51.50)	3,330 (52.05)	747 (48.34)	
Male	3,905 (48.50)	3,045 (47.95)	860 (51.66)	
Race (%)				<0.001
Mexican American	1,060 (8.26)	798 (7.99)	262 (9.76)	
Other races	2,034 (14.09)	1,636 (13.96)	398 (14.79)	
Non-Hispanic White	3,227 (66.99)	2,677 (67.87)	550 (61.96)	
Non-Hispanic Black	1,661 (10.67)	1,264 (10.18)	397 (13.49)	
Education Level (%)				<0.001
Less than high school	1,634 (13.73)	1,178 (12.63)	456 (20.05)	
High School Grad/GED	1,770 (22.35)	1,383 (21.75)	387 (25.79)	
More than high school	4,578 (63.92)	3,814 (65.62)	764 (54.16)	
PIR [mean (SD)]	2.95 ± 1.65	2.99 ± 1.65	2.74 ± 1.61	<0.001
BMI [mean (SD)]	29.32 ± 7.00	28.61 ± 6.61	33.37 ± 7.76	<0.001
Physical activity (%)				<0.001
High	1,838 (25.95)	1,677 (28.66)	161 (10.39)	
Low	4,022 (45.42)	3,030 (42.99)	992 (59.34)	
Medium	2,122 (28.63)	1,668 (28.35)	454 (30.28)	
Smoking status (%)				<0.001
Current	1,573 (18.45)	1,316 (19.10)	257 (14.69)	
Former	1,928 (25.31)	1,392 (23.58)	536 (35.27)	
Never	4,481 (56.24)	3,667 (57.32)	814 (50.04)	
Alcohol intake [mean (SD)]	9.39 ± 22.19	9.98 ± 22.38	5.97 ± 20.72	<0.001
Energy intake [mean (SD)]	2,106.38 ± 824.06	2,129.75 ± 822.33	1,972.38 ± 821.42	<0.001
Hypertension (%)	3,452 (38.61)	2,324 (33.38)	1,128 (68.63)	<0.001
Dyslipidemia (%)	2,149 (25.91)	1,306 (20.90)	843 (54.64)	<0.001
GLU	107.38 ± 30.91	99.05 ± 9.60	155.20 ± 56.76	<0.001
HbA1c	5.64 ± 0.97	5.38 ± 0.37	7.16 ± 1.69	<0.001
CDAI	0.15 ± 4.03	0.25 ± 4.14	−0.40 ± 3.26	<0.001

Data are presented as frequencies (percentages) or mean ± SD. CDAI, composite dietary antioxidant index; PIR, poverty income ratio; BMI, the body-mass index; DM, diabetes mellitus; GLU, blood glucose; HbA1c, glycosylated hemoglobin.

### 3.2 Association between dietary antioxidant intake and DM

In the multivariate adjusted logistic regression model (Model 3) presented in Table 2, we observed that, using vitamin A intake in the lowest quartile (Q1) as the reference, higher quartiles (Q2: OR 1.38 [95% CI 1.01, 1.89]; Q3: OR 1.32 [95% CI 1.00, 1.74]; Q4: OR 1.37 [95% CI 1.03, 1.80]) were associated with an increased risk of diabetes. Conversely, using zinc intake in the lowest quartile (Q1) as the reference, higher quartiles (Q2: OR 0.78 [95% CI 0.57, 1.05]; Q3: OR 0.82 [95% CI 0.64, 1.04]; Q4: OR 0.72 [95% CI 0.52, 1.01]) were associated with a decreased risk of diabetes. Although these findings did not reach statistical significance (*p* = 0.088), a decreasing trend in risk with increasing zinc intake was noted. Furthermore, there was a significant overall reduction in the risk of developing DM with increasing vitamin C intake (*p* = 0.047).

**Table 2:** Results of a multiple logistic regression analysis of the correlation between antioxidant indicators and DM, weighted.

Characteristic		OR (95%CI)	
	Model 1	Model 2	Model 3
**Vitamin A intake**			
Q1	1.00 (Ref.)	1.00 (Ref.)	1.00 (Ref.)
Q2	1.38 (1.08, 1.77)	1.35 (1.01, 1.79)	1.38 (1.01, 1.89)
Q3	1.18 (0.94, 1.49)	1.13 (0.88, 1.45)	1.32 (1.00, 1.74)
Q4	1.05 (0.82, 1.35)	1.04 (0.79, 1.37)	1.37 (1.03, 1.80)
P for trend	1.000	0.911	0.040
**Vitamin C intake**			
Q1	1.00 (Ref.)	1.00 (Ref.)	1.00 (Ref.)
Q2	1.00 (0.83, 1.21)	0.92 (0.75, 1.14)	1.03 (0.80, 1.33)
Q3	0.98 (0.77, 1.25)	0.84 (0.64, 1.09)	0.93 (0.69, 1.25)
Q4	0.70 (0.56, 0.88)	0.58 (0.44, 0.76)	0.73 (0.52, 1.01)
P for trend	0.003	<0.001	0.047
**Vitamin E intake**			
Q1	1.00 (Ref.)	1.00 (Ref.)	1.00 (Ref.)
Q2	0.99 (0.78, 1.25)	1.03 (0.81, 1.33)	1.12 (0.86, 1.45)
Q3	0.72 (0.56, 0.93)	0.78 (0.59, 1.04)	0.89 (0.66, 1.20)
Q4	0.66 (0.52, 0.83)	0.75 (0.56, 1.00)	0.94 (0.66, 1.34)
P for trend	<0.001	0.020	0.464
**Carotenoid intake**			
Q1	1.00 (Ref.)	1.00 (Ref.)	1.00 (Ref.)
Q2	1.29 (1.01, 1.65)	1.28 (1.0, 1.66)	1.31 (1.02, 1.70)
Q3	1.18 (0.94, 1.48)	1.02 (0.79, 1.32)	1.12 (0.85, 1.46)
Q4	0.96 (0.78, 1.19)	0.85 (0.68, 1.07)	1.03 (0.81, 1.32)
P for trend	0.523	0.051	0.567
**Selenium intake**			
Q1	1.00 (Ref.)	1.00 (Ref.)	1.00 (Ref.)
Q2	0.97 (0.80, 1.18)	1.03 (0.84, 1.27)	1.07 (0.83, 1.37)
Q3	0.77 (0.60, 1.00)	0.89 (0.66, 1.19)	0.94 (0.64, 1.39)
Q4	0.82 (0.66, 1.01)	1.01 (0.79, 1.30)	1.21 (0.83, 1.76)
P for trend	0.018	0.765	0.474
**Zinc intake**			
Q1	1.00 (Ref.)	1.00 (Ref.)	1.00 (Ref.)
Q2	0.74 (0.57, 0.95)	0.76 (0.58, 1.0)	0.78 (0.58, 1.05)
Q3	0.70 (0.59, 0.84)	0.75 (0.62, 0.91)	0.82 (0.64, 1.04)
Q4	0.65 (0.52, 0.82)	0.74 (0.57, 0.96)	0.72 (0.52, 1.01)
P for trend	<0.001	0.023	0.088

OR, Odds ratio; CI, Confidence interval; DM, Diabetes mellitus. Model 1: adjusted for no covariates. Model 2: adjusted for basic characteristics (age, gender, race and education level). Model 3: adjusted for all covariates.

### 3.3 Association of CDAI and DM

**Figure 2:**
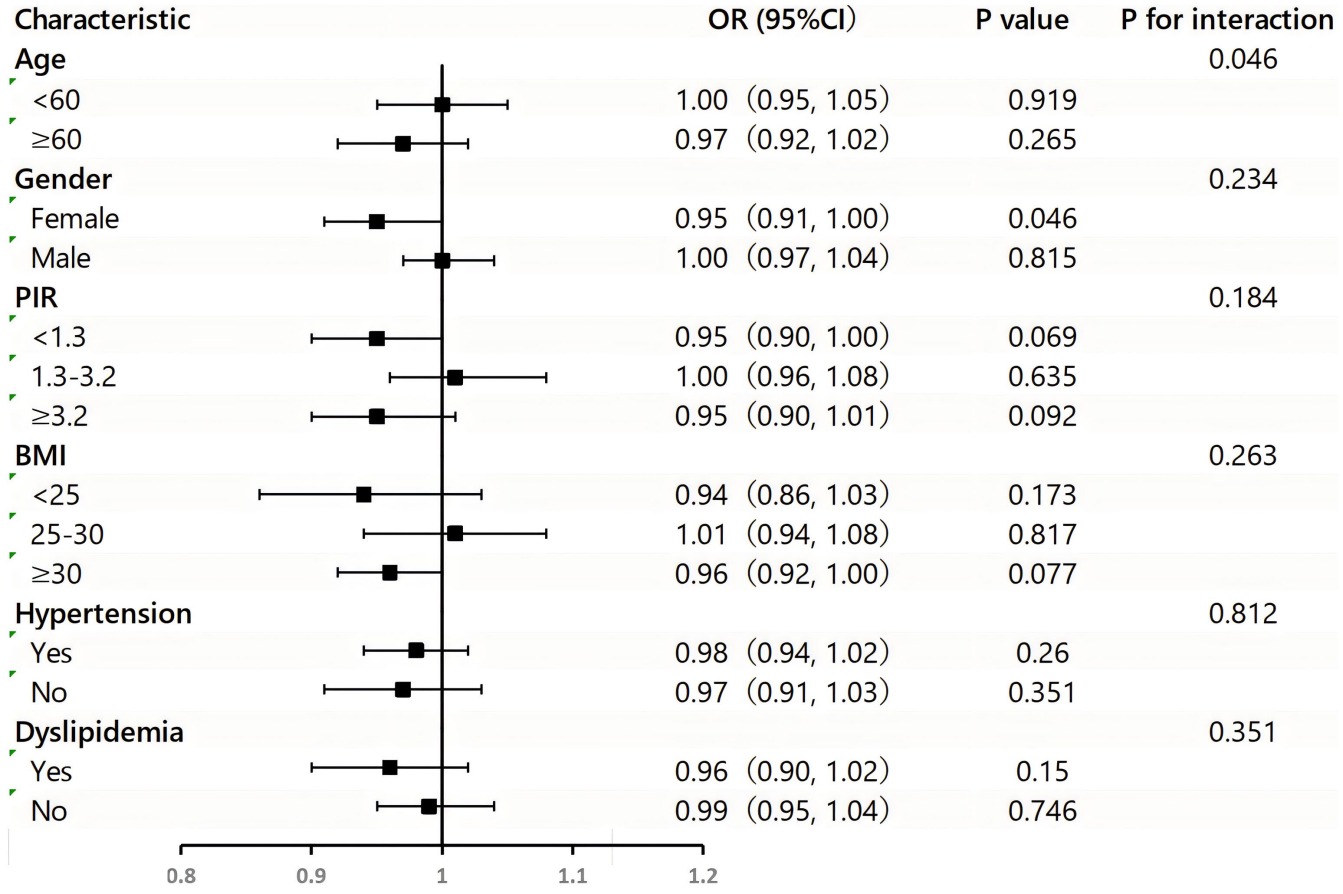
Subgroup analysis for the association between the CDAI and DM.

Higher levels of the Composite Dietary Total Antioxidant Index (CDAI) were associated with a lower risk of DM when CDAI was treated as a continuous variable. This association was statistically significant in both Model 1 (*p* < 0.001) and Model 2 (*p* = 0.003) (Table 3). Additionally, when CDAI was categorized, using the lowest quartile (Q1) as the reference, the results for higher quartiles were consistent (Q2: OR 0.99 [95% CI 0.80, 1.21]; Q3: OR 0.80 [95% CI 0.62, 1.02]; Q4: OR 0.75 [95% CI 0.58, 0.96]). There was an overall trend of decreasing DM risk with increasing CDAI (P for trend = 0.008). However, this association was not significant in Model 3. The restricted cubic spline (RCS) plot from Model 3, which adjusts for all covariates, shows no significant evidence of a non-linear relationship between the Composite Dietary Antioxidant Index (CDAI) and diabetes mellitus (DM) risk (P for non-linearity = 0.827). The curve remains close to the null value of 1.00 across the full range of CDAI values, suggesting a linear and consistent association, with no notable deviations or threshold effects (Figure 2).

**Table 3:** Association of the composite dietary antioxidant index and DM.

Characteristic		OR (95% CI)	
	Model 1	Model 2	Model 3
**Continuous**			
	0.95 (0.93, 0.97)	0.96 (0.93, 0.99)	0.97 (0.94, 1.01)
*p*-value	<0.001	0.003	0.133
**Categories**			
Q1	1.00 (Ref.)	1.00 (Ref.)	1.00 (Ref.)
Q2	1.01 (0.85, 1.20)	0.99 (0.80, 1.21)	1.11 (0.87, 1.41)
Q3	0.75 (0.61, 0.93)	0.80 (0.62, 1.02)	0.94 (0.68, 1.30)
Q4	0.68 (0.54, 0.84)	0.75 (0.58, 0.96)	0.96 (0.68, 1.37)
P for trend	<0.001	0.008	0.619

OR, Odds ratio; CI, Confidence interval; DM, diabetes mellitus; CDAI, composite dietary antioxidant index. Model 1: adjusted for no covariates. Model 2: adjusted for basic characteristics (age, gender, race and education level). Model 3: adjusted for all covariates.

### 3.4 Subgroup analysis

**Figure 3:**
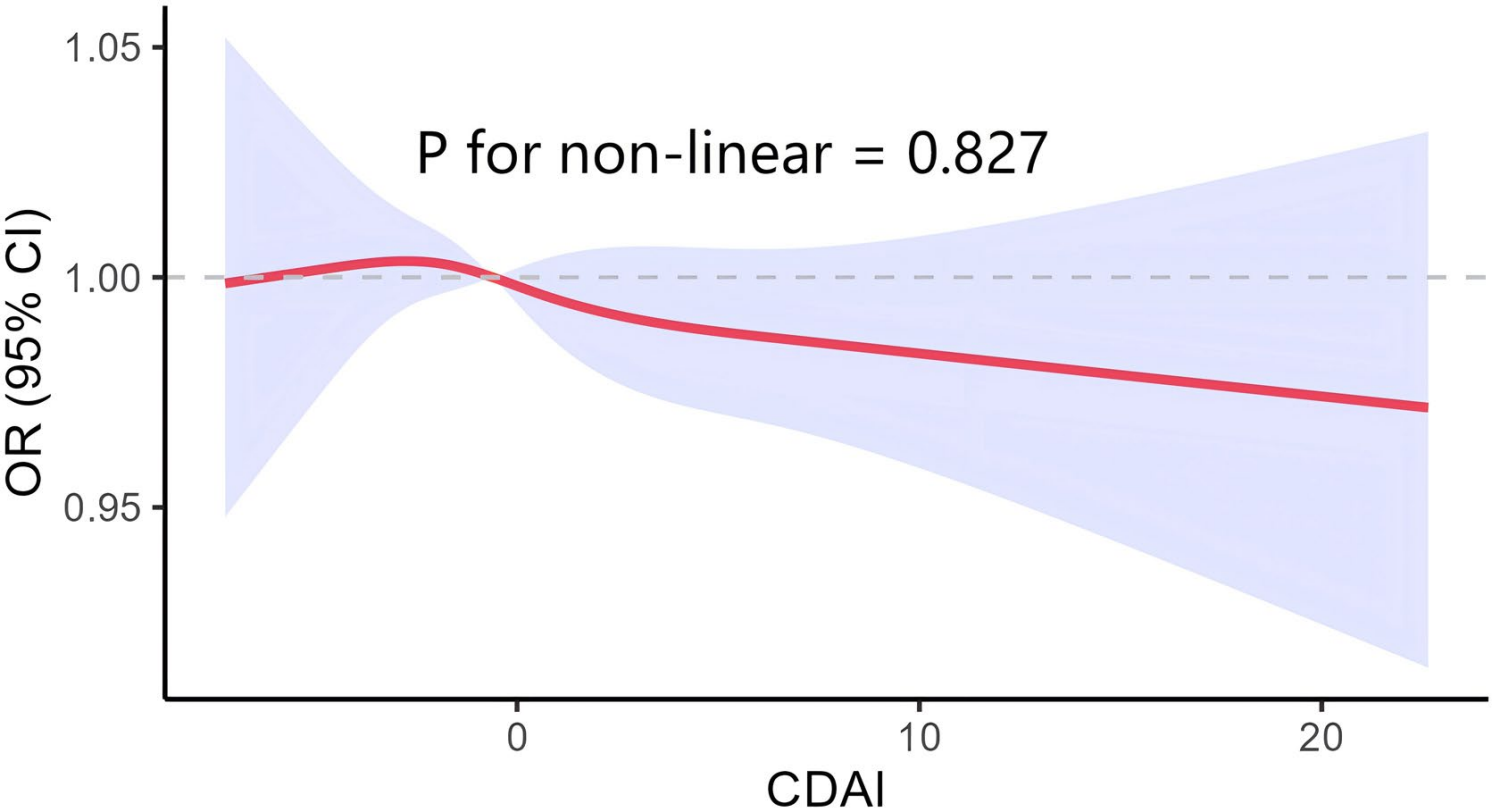
Subgroup analysis for the association between the CDAI and DM.

Figure 3 presents the results of subgroup analyses and interactions, visualized in a forest plot. The association between CDAI and DM was more pronounced in females (*p* = 0.046) after stratification by age, gender, BMI, poverty index, hypertension, and dyslipidemia. Notably, there was an interaction between CDAI and DM for the age covariate (p = 0.046).

## 4 Discussion

To our knowledge, this is the first cross-sectional study examining the relationship between dietary antioxidant intake, CDAI, and diabetes mellitus (DM). We analyzed data from four NHANES cycles (2011–2018) and found that increased intake of vitamin C was associated with a reduced risk of DM. Notably, the negative correlation between CDAI and DM was significant only in the female population, suggesting that a diet high in vitamin C and other antioxidants may be crucial in preventing diabetes, especially among adult women.

There is substantial evidence indicating that oxidative stress plays a critical role in the pathogenesis of diabetes. Excessive production of free radicals and diminished antioxidant defenses lead to increased lipid peroxidation and the development of insulin resistance (25). In pre-diabetes and type 2 diabetes, insulin resistance emerges as a pivotal influencing factor (26).

While several studies have examined the relationship between single dietary antioxidants and type 2 diabetes, such as vitamin C, which may inhibit or ameliorate oxidative stress and insulin resistance, thereby protecting against the development of diabetes mellitus (DM) (6), our findings indicate that increased vitamin C intake correlates with a decreased risk of DM. Additionally, early NHS cohort studies have shown that higher dietary zinc intake is associated with a reduced risk of developing type 2 diabetes mellitus (T2DM) in subsequent years (27). Our findings suggest a similar potential; however, more comprehensive studies are required to elucidate this correlation. However, our study diverges from previous conclusions regarding vitamin A intake.

Vitamin C is a water-soluble antioxidant found in biological fluids (28). Numerous human *in vivo* studies have examined the effects of vitamin C supplementation on markers of lipid, protein, and DNA oxidation, both in the presence and absence of oxidative stress. Although the findings from these studies are generally mixed, evidence suggests that vitamin C supplementation reduces markers of lipid peroxidation (e.g., malondialdehyde and F2-isoprostanes), DNA oxidation (e.g., 8-oxoguanine), and protein oxidation (e.g., nitrotyrosine and protein carbonyls) in biological samples such as plasma, serum, and urine (29–32).Vitamin C also appears to play a crucial role in protecting cells from oxidative damage by accumulating in mitochondria (28). Zinc is essential for insulin crystallization and signaling (33), specifically promoting the activation of the PI3K/Akt pathway, which is vital for glucose metabolism (34). Additionally, zinc acts as a cofactor in antioxidant defense and carbohydrate metabolism (35, 36).

Given the complexity of diet, it is more appropriate to examine the combined effects of antioxidants. Therefore, we introduced the CDAI indicator. Previous studies have shown that high CDAI levels are associated with a reduced risk of diseases such as COPD, hyperlipidemia, and colorectal cancer (17, 37, 38). Additionally, one study found a negative correlation between CDAI and inflammatory cytokines, including IL-1b and TNF-a (39). Another study reported similar associations between CDAI and inflammatory markers like leukocytes and C-reactive protein (17). Oxidative stress can elevate inflammatory factors (40, 41), and inflammation is a known risk factor for DM development (42, 43). Therefore, we hypothesize that dietary antioxidants may mitigate the inflammatory response induced by oxidative stress by modulating leukocytes, C-reactive protein, and other inflammatory markers, thus reducing the risk of DM. This potential mechanism requires further exploration.

Only one previous study has examined the relationship between CDAI and diabetes, showing a negative correlation (18). However, this is not entirely consistent with our findings, which demonstrated a strong correlation only in adult women. The discrepancy may be attributed to differences in CDAI calculation methods. The previous study used a pooled score incorporating six dietary antioxidants (vitamins A, C, and E, manganese, selenium, and zinc) (15, 18). As the current dietary module of the NHANES database lacks information on dietary manganese, our study employed modified versions of vitamin A, vitamin C, vitamin E, carotenoids, selenium, and zinc to calculate the Composite Dietary Antioxidant Index (CDAI) (21). Dietary manganese deficiency can lead to increased ROS production and oxidative stress (44), and manganese is vital for normal insulin synthesis and secretion (45). This discrepancy may partly explain the differences in study outcomes.

Due to elevated estrogen levels in women, research has shown that estrogen binds to estrogen receptors and activates the MAP kinase-NF-κB pathway, which subsequently upregulates the expression of antioxidant enzymes (46, 47). Estrogen functions as an antioxidant by enhancing the expression of antioxidant and longevity-related genes. Additionally, estrogens are implicated in inflammation; they interact with their receptors to modulate various inflammatory factors, including cytokines and inducible nitric oxide synthase (48). These factors may further account for why our findings were significant only in the female population. Therefore, additional studies are necessary to further explore the relationship between the Composite Dietary Antioxidant Index (CDAI) and diabetes mellitus (DM).

This study has limitations. First, it was conducted on U.S. adults, excluding populations from other regions where dietary habits may differ. Second, CDAI data were derived from two 24-h dietary recall interviews, subject to recall bias. Lastly, as a cross-sectional study, it cannot establish causality between dietary antioxidant intake, CDAI, and DM, only associations.

## 5 Conclusion

In conclusion, this cross-sectional study suggests that a diet rich in vitamin C may serve as a significant preventive measure against diabetes mellitus (DM) in adults. In the female population, the Composite Dietary Antioxidant Index (CDAI) was strongly associated with the risk of developing DM. Although zinc intake showed a trend toward a lower risk of diabetes, further epidemiological evidence, particularly from prospective studies, is required to confirm these relationships and to develop more accurate and effective preventive and therapeutic strategies for DM.

## Data Availability

Publicly available datasets were analyzed in this study. This data can be found at: https://wwwn.cdc.gov/nchs/nhanes/.
